# *SOX2* and *OCT4* mRNA-Expressing Cells, Detected by Molecular Beacons, Localize to the Center of Neurospheres during Differentiation

**DOI:** 10.1371/journal.pone.0073669

**Published:** 2013-08-27

**Authors:** Mirolyuba Ilieva, Martin Dufva

**Affiliations:** Department of Micro- and Nanotechnology, Technical University of Denmark, Lyngby, Denmark; Baylor College of Medicine, United States of America

## Abstract

Neurospheres are used as *in vitro* assay to measure the properties of neural stem cells. To investigate the molecular and phenotypic heterogeneity of neurospheres, molecular beacons (MBs) targeted against the stem cell markers *OCT4* and *SOX2* were designed, and synthesized with a 2’-O-methyl RNA backbone. *OCT4* and *SOX2* MBs were transfected into human embryonic mesencephalon derived cells, which spontaneously form neurospheres when grown on poly-L-ornitine/fibronectin matrix and medium complemented with bFGF. *OCT4* and *SOX2* gene expression were tracked in individual cell using the MBs. Quantitative image analysis every day for seven days showed that the *OCT4* and *SOX2* mRNA-expressing cells clustered in the centre of the neurospheres cultured in differentiation medium. By contrast, cells at the periphery of the differentiating spheres developed neurite outgrowths and expressed the tyrosine hydroxylase protein, indicating terminal differentiation. Neurospheres cultured in growth medium contained *OCT4* and *SOX2*-positive cells distributed throughout the entire sphere, and no differentiating neurones. Gene expression of *SOX2* and *OCT4* mRNA detected by MBs correlated well with gene and protein expression measured by qRT-PCR and immunostaining, respectively. These experimental data support the theoretical model that stem cells cluster in the centre of neurospheres, and demonstrate the use of MBs for the spatial localization of specific gene-expressing cells within heterogeneous cell populations.

## Introduction

Stem cells are found in most tissues and are characterized by their ability to self-renew and undergo differentiation into specialized effector cells. These properties make stem cells crucial for maintaining tissue homeostasis, and for tissue repair after injury. Stem cells are therefore potentially useful for therapeutic applications. However, stem cell, either transplanted or endogenous can also be involved in pathological processes like carcinogenesis. Stem cells exist in either a quiescent or activated state, and have the ability to switch between these states [1]. The progeny of stem cells have also been shown to have the ability to revert back to stem cells [2].

Neural stem cells (NSCs) are tissue-specific stem cells that have the capacity for proliferation, self-renewal, and production of a large family of differentiated functional progeny [3]. NSCs exist in specialized ‘niches’ in the adult mammalian brain and continuously generate new neurons that functionally integrate into neural circuits [4]. Experimentally, long-term culture systems are based on cell grown as adherent monolayers or as neurospheres. The latter are free floating clonal cell aggregates. *In vitro* growth of NSCs as neurospheres allows for continuous propagation of potentially heterogeneous populations of NSCs and their progenitors. Neurospheres exhibit intra-clonal neural cell-lineage diversity containing, in addition to NSCs, neuronal and glial progenitors at different stages of differentiation [5]. Neurosphere formation assays are widely used as a model for neuronal development, and for studying neurogenesis [6]. They have also been used to characterize the factors and molecular mechanisms controlling stem cell properties, and to find the gene expression signatures that characterize different cell populations [7,8].

However, the following limitations of neurospheres mean that they are insufficient on their own to definitively prove the existence of a stem cell population within the clusters [9,10]. First, multiple populations of more committed progenitor cells, as well as stem cells, can give rise to neurospheres. Second, most of the stem cells are in the quiescent stage, which is incompatible with neurosphere formation. Third, cells within the neurosphere can shuttle between quiescent and activated states, and even more committed progenitors can revert back to a more primitive state [11]. The neurosphere is a dynamic structure and cell-cell or cell-environment interactions may have a significant impact on NSC differentiation, and contribute to the heterogeneity of the neurosphere [12]. It is therefore important to employ time lapse microscopy when using the neurosphere forming assay, in order to accurately and confidently detect cells with stem cell characteristics within the clusters, and track their behavior when exposed to different stimuli.

With these limitations in mind, the following questions arise: do neurospheres contain cells with a stem cell signature; what is the distribution of cells within the clusters (i.e. do they form niches); what is their fate during differentiation; and, most importantly from an experimental point of view, how can cells be tracked in real time without affecting cell viability and differentiation? Although a universal stem cell marker does not exist, one of the most meaningful measures of 'stemness' is the expression of transcription factors such as OCT4 and SOX2. However, the detection of expression of these genes in living cells usually requires fusion of *OCT4* or *SOX2* gene promoters with a reporter gene, such as green fluorescent protein (GFP). Instead of using genetic manipulation, transcription factor gene expression can also be detected using molecular beacon (MB) technology, in which the presences of specific mRNAs are detected after transfection [13–16]. MBs are hairpin oligonucleotides with fluorescent dye on one end, and quencher attached to the other. The sequence within the loop is complementary to the desired target mRNA [17]. When the MB hybridizes to the target, the fluorochrome separates from the quencher, and a signal can be detected. Recently, MBs targeted against *SOX2* mRNA were applied to the sorting of cells isolated from mouse brains [18].

In this study we investigate, as a function of time, the location of *OCT4* and *SOX2* mRNA-positive cells within neurospheres grown in either growth medium (GM) or in differentiation medium (DM). We demonstrate that MBs can be used to determine the exact location of *OCT4/SOX2* positive cells inside living neurospheres, allowing us to confirm a model of stem cell distribution within differentiating neurospheres.

## Materials and Methods

### Cell culture and media

LUHMES (Lund human mesencephalic cell line, ATCC, CRL-2927) is a subclone of the tetracycline-controlled, v-myc-overexpressing human mesencephalic-derived cell line MESC2.10. For neurosphere formation, cells were cultured in cell culture flasks coated with 50µg/ml poly-L-ornitine (Sigma) overnight at room temperature (RT), followed by coating with 1µg/ml human fibronectin (Sigma) in H_2_O for at least 3h at 37°C. Adherent monolayer cells were cultured in cell culture flasks coated with Geltrex^®^, a reduced growth factor basement membrane extract purified from murine Engelbreth-Holm-Swarm tumor (Invitrogen), diluted 1:100 in PBS for 1h at 37°C. Cultures were maintained in growth medium (GM) consisting of Advanced DMEM/F12 (Sigma), 2mM L-glutamine, 1x N2 supplement (Gibco), 1% penicillin/streptomycin, and 40ng/ml recombinant bFGF (Invitrogen), at 37°C in a humidified atmosphere containing 5% CO_2_.

The human embryonic stem cell (hESC) line BG01V (ATCC, SCRC-2002) was propagated on Mitomycin C-treated mouse embryonic fibroblasts (ATCC, MEF SCRC-1040) in complete GM DMEM/F12 containing 2mM L-glutamine, 1x MEM non-essential amino acid solution (Gibco), 0.1mM 2-mercaptoethanol (Sigma), 4ng/ml bFGF (Invitrogen), 5% knockout serum replacement (Invitrogen), 15% fetal bovine serum (FBS), and 1% penicillin/streptomycin. The medium was change daily. Cells were adapted to grow on Geltrex^®^-coated flasks and the final experiments were performed on this cell growth matrix.

#### Differentiation of LUHMES

Differentiation was initiated by adding differentiation medium (DM) consisting of advanced DMEM/F12, 1x N2 supplement, 2mM L-glutamine, 1mM dbcAMP (Sigma), 1µg/ml tetracycline, and 2ng/ml recombinant human GDNF (R&D system). LUHMES cells differentiated into dopaminergic neurons after five days exposure to differentiation medium (DM).

### Molecular beacons

mRNA-targeting MBs were designed using Beacon Designer 7.9 (Premier Biosoft). To avoid cross hybridization and formation of secondary structures, the target sequence for each MB was analyzed using BLAST (http://blast.ncbi.nlm.nih.gov/) and mFOLD (downloadable from http://mfold.rna.albany.edu/). MBs were synthesized with a 2’-O-methyl RNA backbone, a Texas Red molecule attached to the 5’ end, and a black hole quencher 2 (BHQ2) attached to the 3’ end (Eurofins MWG Operon). The sequences of the MBs used were: *OCT4* (NM_203289) - CGCUC UCAUUCACCCAUUCCCUGUU G A G C G ; *SOX2* (NM_003106.3) - CGCUC CGCCGCCGAUGAUUGUUAUUAU G A G C G (underlined sequences indicate the stem formation sequences). MBs were diluted in RNase/DNase free dH_2_O to yield stock 100µM stock solutions, and stored at -20°C.

### Transfection of MBs into LUHMES cells for monolayer growth

MBs were introduced into the cytoplasm of living cells by toxin-based membrane permeabilization using Streptolysin O (SLO, Sigma). Prior to use, SLO (1 µg/ml) was activated with Tris (2-carboxyethyl) phosphine hydrochloride solution (TCEP, Sigma) at a final concentration of 5mM, for at least 30 min at 37°C. Adherent LUHMES cells were washed with Dulbecco’s phosphate-buffered saline (DPBS) without Ca^2+^ and Mg^2+^, and trypsinized for 3 min at 37°C. The cell suspension was centrifuged for 5 min at 190 x g to collect the cells. The cells were re-suspended in Opti-MEM medium. 1 x 10^5^ cells were incubated with activated SLO at a concentration of 17 U/ml (230ng/ml) and MBs (2µM) in a final volume 100µl for approximately 15 min. Permeabilized cells were resealed by washing in DPBS containing Ca^2+^ and Mg^2+^, and plated on Geltrex^®^-coated dishes in GM. The differentiation process was started 24 h after plating by exchanging the GM with DM.

#### Transfection of MBs into neurospheres

1x10^5^ cells were seeded on poly-L-ornitin/fibronectin-coated 12-well plates for neurosphere formation. Neurospheres were transfected 72h after neurosphere formation. Free-floating neurospheres were collected by centrifugation for 5 min at 190 x g, and then incubated with the mixture containing 17U/ml TCEP-activated SLO, and 2µM MBs for 15 min at 37°C.

### Transfection of MBs into hESC

hESCs growing on MEF were treated with collagenase IV solution in DMEM/F12 at a concentration of 0.5 mg/ml (or ~200U/ml) for 45 min. When the majority of the hESC colonies detached, an appropriate volume of DMEM/F12 was added and the cell suspension was collected. Cells were centrifuged for 5 min at 200 x g at 25°C, the cells re-suspended in Opti-MEM, and the procedure for transfection of adherent LUHMES was followed. Finally, cells were plated on Geltrex^®^-coated dishes in complete hESC medium.

### Cell viability assays

Cellular viability after SLO-mediated transfection was detected by calcein/propidium iodide staining. The medium from each well was carefully removed and cells were incubated for 30 min with 3µM calcein AM (live dye), and 2.5µM propidium iodide (dead dye), diluted in warm 1x DPBS without Ca^2+^ or Mg^2+^ prior to fluorescent microscopy.

### Quantitative real-time polymerase chain reaction (qRT-PCR)

Total RNA from adherent cells or neurospheres grown for five days in GM or DM respectively was isolated using the RNeasy Mini kit (Qiagen). Adherent cells were lysed directly on the dish, while neurospheres were collected by centrifugation prior to the addition of the lysis buffer. The lysates were collected and purified according to the manufacturer’s instructions. Single-stranded cDNA was prepared from total RNA using random primers under standard conditions using MultiScribe Reverse Transcriptase (Applied Biosystems). The cDNA from each sample was diluted and used for qRT-PCR analysis based on the Taqman assays (Invitrogen) quantifying *OCT4* (ID Hs04260367_gH), *SOX2* (ID Hs01053049_s1), *TH* (ID Hs00165941_m1) with *GAPDH* (ID Hs03929097_g1) used as an internal positive control. qPCR amplifications were performed in duplicates using the Chromo4 Real-Time PCR Detection system (BioRad) at 95°C for 10s, followed by 40 cycles of 95°C for 5s and 60°C for 30s. In order to quantify the relative expression of the gene, the C_t_ (threshold cycle) values were normalized to the C_t_ value of GAPDH (e.g ΔC_t_ = C_t_(Oct 4)-C_t_(GAPDH)). All experiments included negative controls of no cDNA in the reaction mixture.

### Immunocytochemistry

Neurospheres were grown in 8-well LabTek Permanox chamber slides (Nunc, Roskilde, Denmark), precoated with poly-L-ornitin/fibronectin, while monolayers were cultured on Geltrex^®^-coated chamber slides. Cells were fixed in 4% (v/v) formaldehyde in PBS containing 0.05M sucrose (v/v) and 0.4mM CaCl_2_ for 20 min, and blocked with 1% (w/v) bovine serum albumin (BSA) in PBS. An overnight incubation with primary antibody diluted in PBS containing 5% (v/v) fetal calf serum (FCS), 50mM glycine, and 0.025% (v/v) Triton X100, was followed by an incubation with secondary antibody for 1h at RT. The primary and secondary antibodies used in this study are listed in Table 1. DAPI (0.1 µg/ml) was added to the buffer in the last washing step in order to stain the nuclei.

**Table 1:** The primary and secondary antibodies used within this study.

Antibody	Host	Working dilution	Source	Cat No.
Primary antibodies				
OCT4	Rabbit	1:400	Cell Signaling Technology	#2750
SOX2	Goat	1:100	Santa Cruz Biotechnology, Inc.	Sc-17320
TH	Rabbit	1:100	Cell Signaling	#2792
Secondary antibodies				
Alexa Fluor 488 anti-rabbit IgG	Goat	1:1000	Life Technologies	A-11008
FITC-conjugated anti-goat IgG	Rabbit	1:40	Dako Cytomation	F025002

### Imaging and image analysis

Phase contrast and fluorescent images were acquired on a Carl Zeiss Axio Vision 4.8.2 microscope equipped with the ApoTome imaging system, 10x/0.3 and 40x/0.75 Plan-Neofluar objective, HXP lamp, and a Zeiss Axiocam MRm B/W camera. The same exposure time and filter set (43 HE Ds Red 538-570 nm) were used for all experiments.

Single-cell image analysis was performed using ImageJ software (http://rsbweb.nih.gov/ij/). Regions of interest (ROI) were draw around cells and the total fluorescence intensity was subsequently determined. The background fluorescence was measured by drawing a ROI in an area outside the cells of interest. The background fluorescent intensity was subtracted from the cellular measurement as previously described [19].

### Statistical analysis

Results are expressed as mean ± standard error of the mean (S.E.M.). qRT-PCR results were analyzed using a Student’s t-test (n=5). The data are expressed as a percentage of the highest value of the group, with the highest value set to 100%. Analyses were performed using GraphPad Prism v6 (GraphPad Software Inc., CA, USA).

## Results

### LUHMES-derived neurospheres express both stem cell markers OCT4 and SOX2

**Figure 1:**
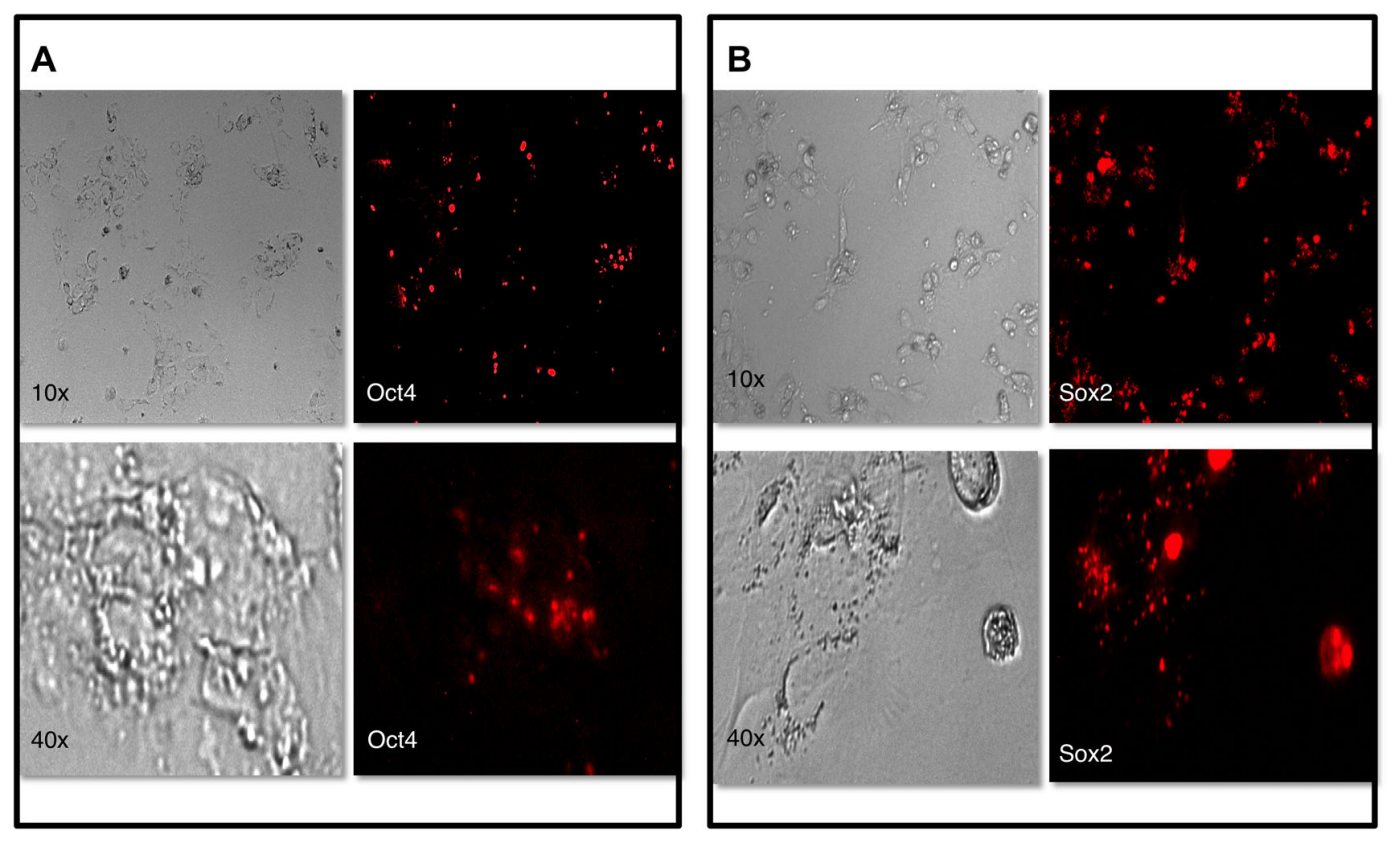
Detection of *OCT4* and *SOX2* mRNA expression in human embryonic stem cells BG01V. A: OCT4 mRNA expression and B: SOX2 expression.

According to the original description by Lotharius et al. (the depositor of the LUHMES cell line in ATCC), LUHMES cells grow as a monolayer on plates pre-coated with poly-L-ornitine/fibronectin [20]. Under these conditions, we observed consistently that the cells detached and formed free-floating neurospheres. By contrast, LUHMES cells grown on Geltrex^®^ formed a monolayer. We therefore had the means to study LUHMES cells in both monolayer and neurosphere configurations. MBs targeting *OCT4* and *SOX2* mRNAs were designed, synthesized with a 2’-O-methyl RNA backbone and used to study the expression patterns in monolayers and neurospheres respectively. The utility of MBs targeting *OCT4* and *SOX2* was first verified by introducing them into a hESC cell line, which is known to highly express *OCT4* and *SOX2 mRNA*, and is therefore a good positive control for the method. Cells were examined 24h after transfection and signals from both beacons were detected (Figure 1). MBs were next introduced into LUHMES cells by SLO-mediated membrane permeabilization. This transfection method did not adversely affect cell viability of neurospheres as determined by calcein/propidium iodide staining (data not shown). Overall, there were very low numbers of apoptotic cells and most of the dead cells were found outside the neurospheres. Apoptosis and necrosis was not observed in the inner core of the neurospheres. In addition to demonstrating that MBs do not promote cell death, this analysis also confirmed that fluorescence inside the neurospheres is specific for MB’s and not auto-fluorescence from dead or dying cells. Monolayers of LUHMES showed >95% viability after the transfection procedure (data not shown).

**Figure 2:**
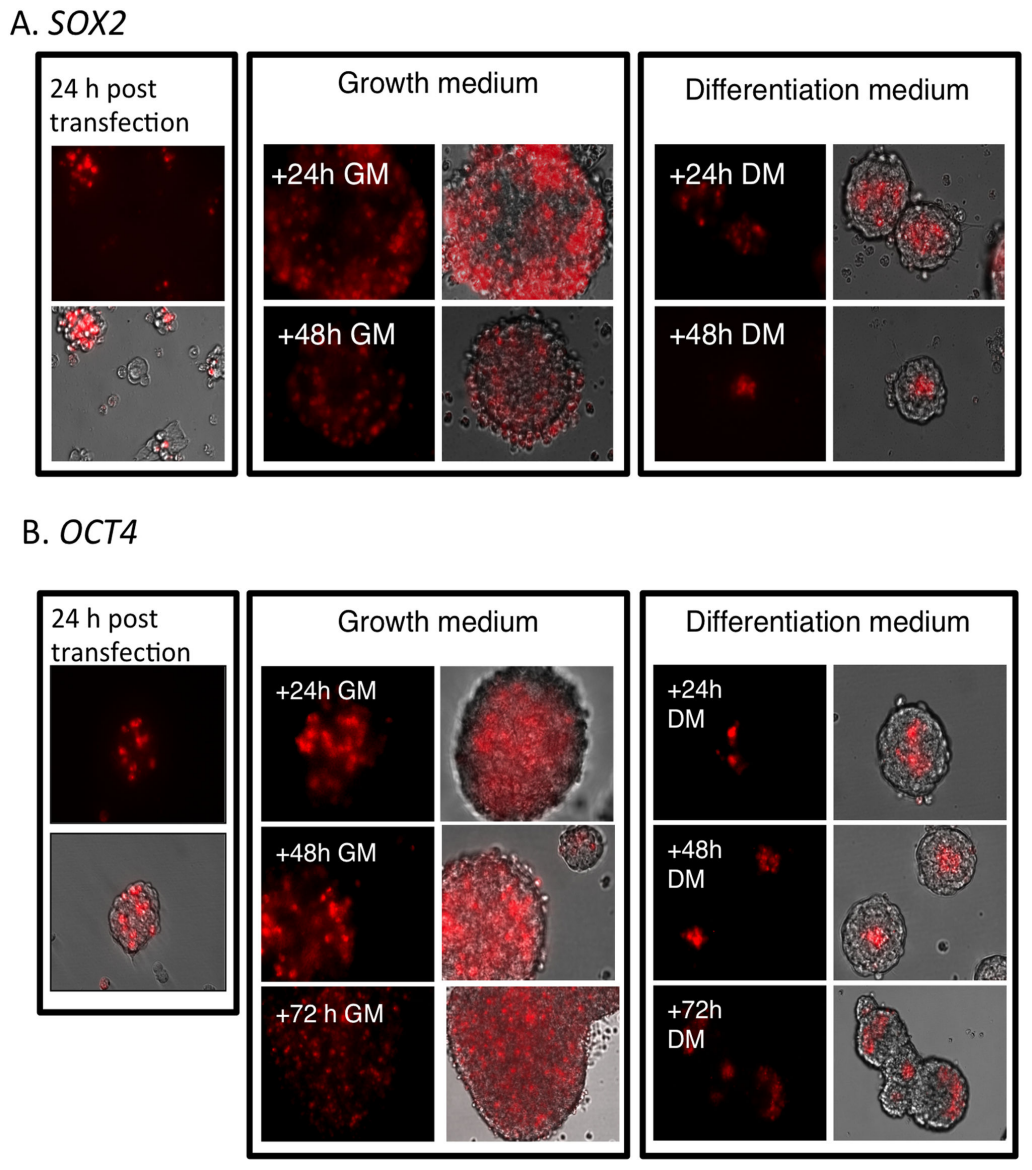
Using MBs to detect stem cell mRNA markers in neurospheres. *SOX2* (A) and *OCT4* (B) mRNA expression were detected with SOX2-MB and OCT4-MB respectively. Cells were kept in GM until 24h post-transfection (left panels in Figures A and B respectively), at which time the medium was switched to either GM (middle panels) or DM (right panels). Gene expression was detected by microscopy as indicated in the figure.

24h after SLO transfection, cells positive for *SOX2* and *OCT4* were localized on the surface of the neurospheres (Figure 2A and B, left panels). The preference for this localization pattern is likely due to transfection being more efficient in cells localized on the surface of the neurospheres compared to cells in the middle of the neurospheres. Cells positive for *SOX2* and *OCT4* mRNA, respectively, were found exclusively in the center of the spheres after 24h culture in DM (48 h after transfection, Figure 2A and B, right panels). By contrast, cells positive for *SOX2* and *OCT4*, were diffusely spread within the neurospheres after 48h culture in GM (48 h after transfection, figure 2A and B, middle panels). Both the number of *OCT4* and *SOX2*-positive cells, and the absolute number of cells, increased with time in the spheres grown in GM. It was difficult to determinate the number of the respective *OCT4* and *SOX2* positive cells within neurospheres in DM because the cells formed tight clusters in the center.

**Figure 3:**
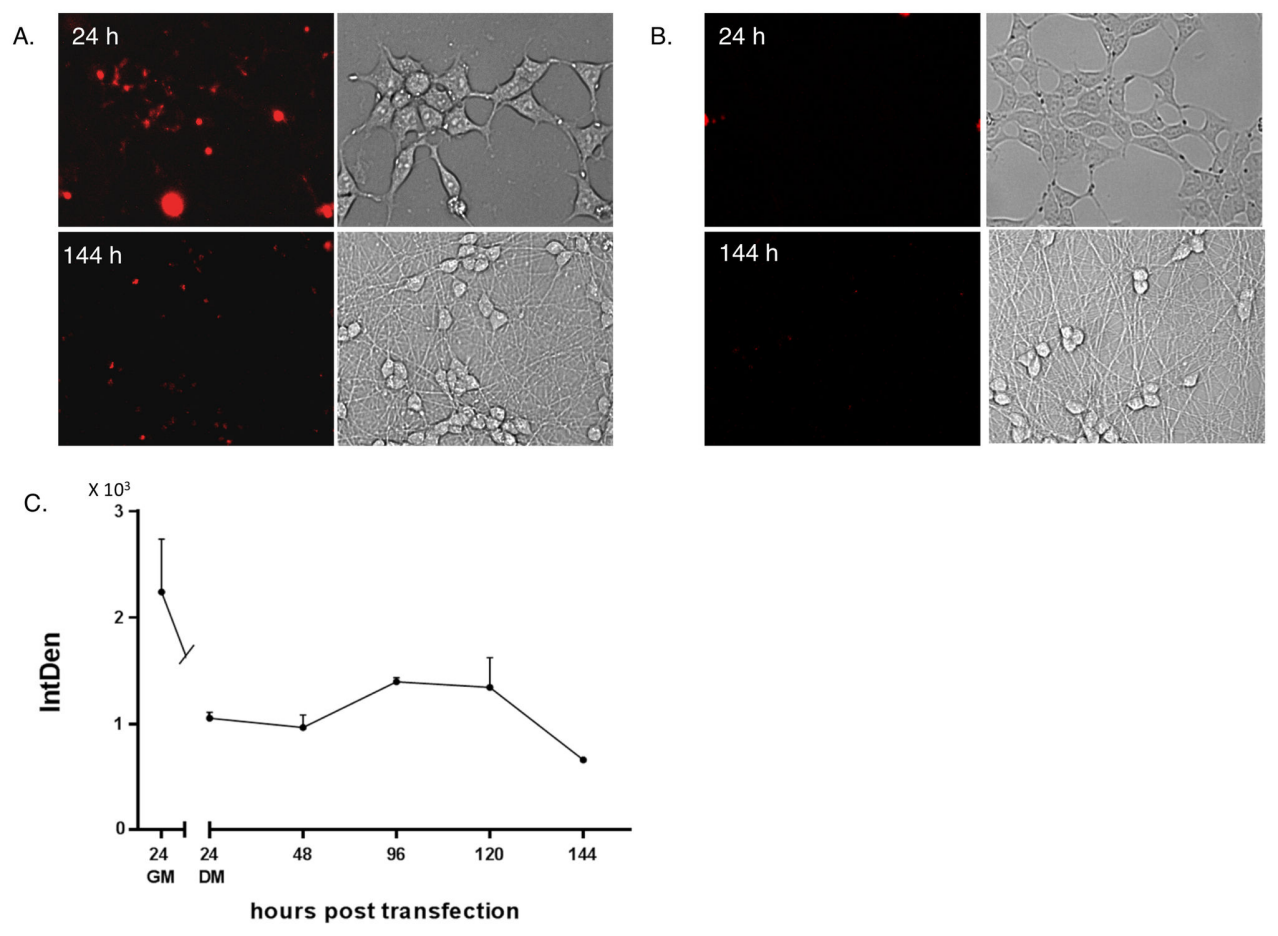
Detection of SOX2 and OCT4 mRNA in adherent LUHMES cells. LUHMES cells were transfected with SOX2-MB (A) or OCT4-MB (B). Bright field and fluorescent photomicrographs were taken 24h after transfection and 144h after induction of differentiation. **C**. Integrated density of *SOX2* targeting MBs as function of time. Data is presented as mean ± S.E.M.

LUHMES cells grown as adherent monolayers expressed *SOX2* but not *OCT4* mRNA (Figure 3). The *SOX2*-MB signals were quantified by image analysis and showed that the MB-SOX2 signal was high 24h after transfection, and decreased 24h after the start of the differentiation process. The MB-SOX2 signal intensity fluctuated during culture in DM, and was halved after 144 h compared to initial values. At 144h post induction of differentiation, LUHMES cells displayed a clear neuronal morphology.

### Using MB to track OCT4 mRNA expressing cells inside neurospheres during differentiation

**Figure 4:**
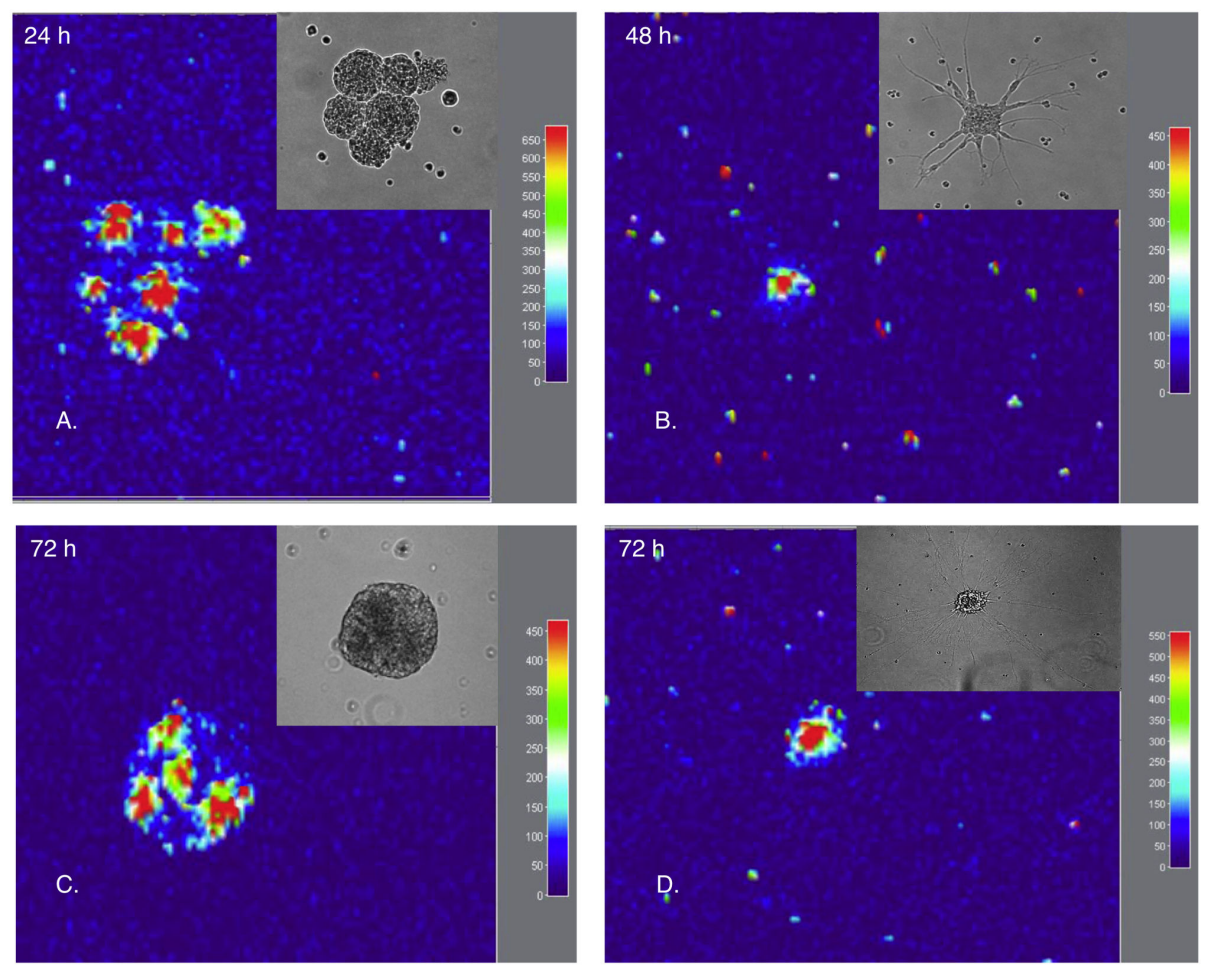
Tracking *OCT4*-positive cells inside neurospheres during differentiation. **A**. 24h after initiation of differentiation, *OCT4*-positive cells are still diffusely spread in some neurospheres, while in others they start localizing to the inner core. **B**. 48h later the neurospheres attach to the matrix and the cells located around the rim start to form neurites. Neurospheres demonstrate varying sizes and differentiation potential. **C**. Fusion between neurospheres was seen, together with neurite formation **D**. In both cases, the MB-detected *OCT4*-positive cell population was located in the middle of neurospheres. Images were acquired with ApoTome Imaging system, 10x/0.3 Plan Neofluar objective and DsRed filter set.

*OCT4* mRNA expression was followed over time in neurospheres during the process of differentiation. The images of the neuropheres were captured as z-stacks with a Zeiss Apotome and assembled into 3D representations of the *OCT4* expressing cells. As mentioned before, some of the neurospheres attached to the bottom of the plate when exposed to DM. The neurospheres were of varying size from small (50µm in diameter) to large (200µm in diameter). Cells located at the rim of the sphere formed long thin neurites and differentiated into cells with neuronal morphology (Figure 4B and 4D inset). Other neurospheres grew in size instead of showing signs of differentiation (Figure 4C inset). Fusion between growing neurospheres was also seen (Figure 4A inset). In all cases, after switching to DM, *OCT4*-mRNA positive cells were organized in the center of the differentiating neurospheres (Figure 4B, D) or in the center of every individual fusing neurosphere (Figure 4 C). The cells at the periphery of some neurospheres showed clear evidence of neural outgrowths, indicating that these cells differentiated under these conditions.

### qRT-PCR and immunocytochemical analysis of stem cell and neurones

**Figure 5:**
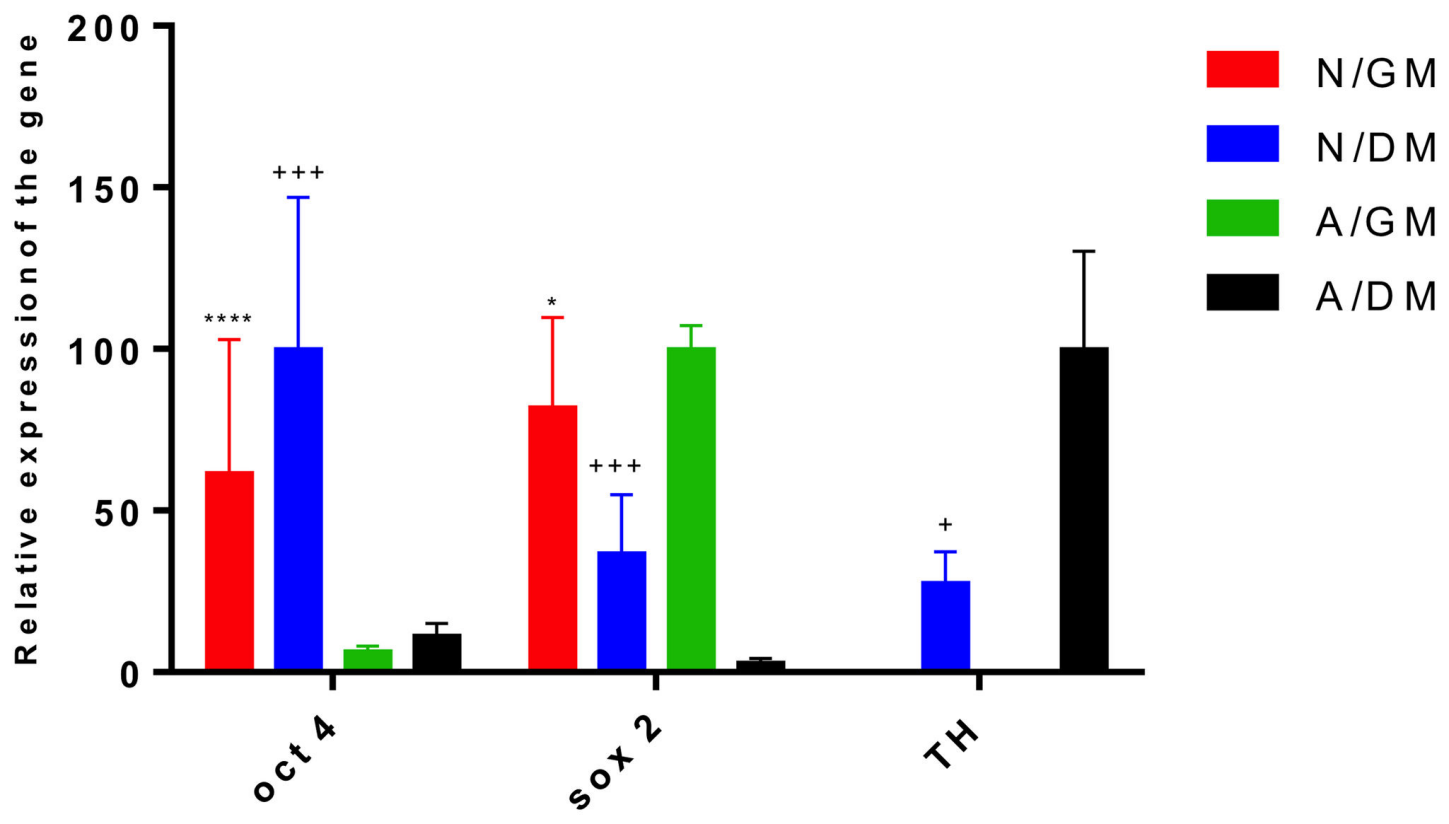
The relative gene expression of *OCT4*, *SOX2* and the neuronal marker *TH* mRNA under different growth conditions. Cells grown as neurospheres in GM (red bar, N/GM), cells grown as neurospheres in DM (blue bar, N/DM), cells grown as monolayer in GM (green bar, A/GM), and cells grown as monolayer in DM (black bar, A/DM) for 5 days. Expression was normalized to GAPDH mRNA expression. Results are expressed as mean ± S.E.M. as a percentage of the highest value in the group set to 100%. n = 5 *p<0.05, ****p<0.0001 neurosphere in GM compared to monolayer cell growth in GM, +p<0.05 +++p<0.001 neurospheres in DM compared to monolayer cells cultured in DM.

qRT-PCR confirmed that neurospheres expressed both *SOX2* and *OCT4* mRNA (Figure 5). No significant differences in *OCT4* expression levels were found between growing and differentiated neurospheres. The *OCT4* expression was barely detectable in either differentiating or growing LUHMES cells grown as monolayers. In contrast, significant *SOX2* expression was detected in both neurospheres and adherent cells in GM. The *SOX2* expression was dramatically decreased during differentiation of adherent cells and neurospheres, the latter growth condition showing a less pronounced decrease in *SOX2* expression. Both *SOX2*-MB and qRT-PCR were therefore able to detect decreases in the *SOX2* expression. Cells grown in GM as neurospheres or as monolayer did not expressed *TH*. By contrast, significant difference in *TH* mRNA expression was detected between cells in DM grown as neurospheres or as monolayer respectively. The highest increase of TH expression was found in monolayer cultures as compared to the neurosphere cultures (Figure 5).

**Figure 6:**
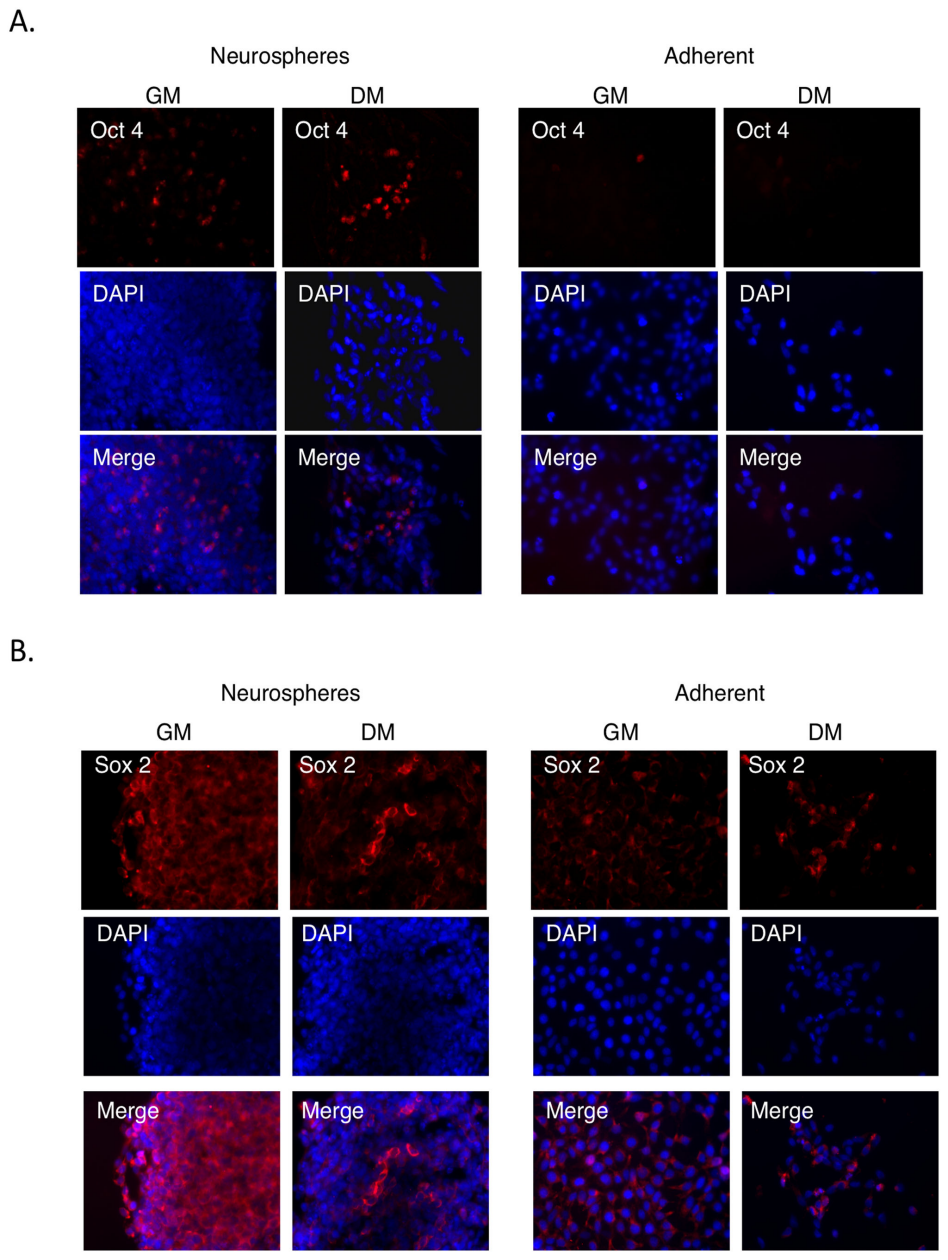
Expression pattern of OCT4 and SOX2 protein in neurospheres. **A**. Immunostaining with an OCT4 antibody. Expression at the protein level was detected in neurospheres under both GM and DM culture conditions. OCT4-positive cells are diffusely spread within neurospheres when cultured in GM, and localized in the center of neurospheres in DM. OCT4 expression at the protein level was not detected in cells growing as a monolayer. **B**. Immunostaining with a SOX2 primary antibody. The distribution of SOX2-positive cells inside neurospheres in DM mirrored the distribution of MB-detected SOX2-positive cells, being inner core localized. Adherent cells cultured in GM highly expressed SOX2 with cytoplasmic localization of the protein. During differentiation, protein levels decreased, which positively correlated with the data from qRT-PCR.

Staining with antibodies targeted against OCT4 demonstrated protein expression in neurospheres, but not in monolayer cultures (Figure 6A), corroborating the qRT-PCR and MB results. Moreover, the OCT4 protein was localized in the cytoplasm of the neurosphere cells cultured in differentiation medium. Due to the high cell number in neurospheres grown in GM, it was difficult to determine the intracellular localization of the OCT4 protein under this condition. It has been described previously [21,22] that two splice variants of OCT4 exist, OCT4A and OCT4B, with nuclear and cytoplasmic localization domains, respectively. OCT4A is restricted to stem cells, whereas OCT4B can be detected also in various non-pluripotent cell types [23]. The antibody used in this study detects both isoforms.

**Figure 7:**
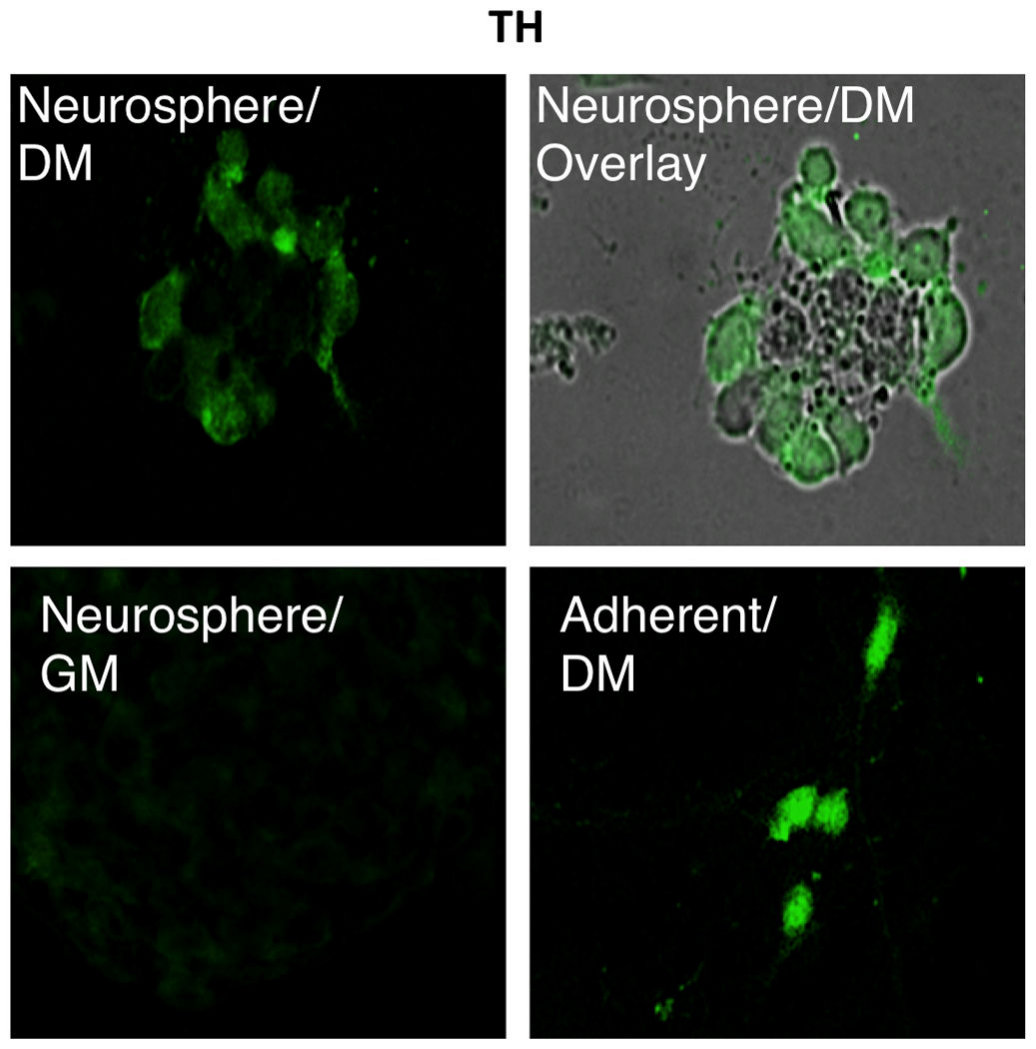
Immunostaining for TH protein. TH expression was not detected in cell in neurospheres in GM. TH positive cells were localized on the rim of the neurospheres in DM whereas the inner core localized cells showed to be negative for expression of the neuronal marker. Differentiated LUHMES cells grown as monolayer were used as positive control.

The SOX2 protein was detected and had cytoplasmic localization under all growth conditions (Figure 6B). A decrease in the number of SOX2 protein-positive cells was observed in both monolayer cultures and neurospheres cultures after exposure to DM. These results again corroborate the qRT-PCR and *SOX2*-MB results. OCT4 and SOX2 protein positive cells were localized in the center of neurospheres grown in DM. By contrast, cells positive for TH were localized at the rim of the neurospheres whereas the cells in the inner core seem to be negative for this neuronal marker (Figure 7). Differentiated cells at the rim of the neurospheres showed less prominent TH expression than differentiated monolayers of cells (Figure 7). Non-differentiated cells in neurospheres or as monolayers did not display any TH protein expression (Figure 7 and data not shown).

## Discussion

By applying MB-technology for the detection of *SOX2* and *OCT4* mRNA in individual cells, we visualized a population of cells displaying a stem cell-like gene expression in LUHMES-derived neurospheres. *OCT4* and *SOX2* mRNA-positive cells were found in the center of the spheres cultured in DM, while cells in the periphery showed signs of neuronal development. *SOX2* was also expressed in monolayers of LUHMES cells and *SOX2* mRNA expression decreased upon differentiation. By contrast, LUHMES cells grown as monolayer did not demonstrate significant *OCT4* expression, suggesting that cells growing as neurospheres are subjected to different microenvironmental conditions, such as differences in growth factor levels and pO_2_. This change of the environment appears to induce *OCT4* mRNA expression in the LUHMES cells. It is however unclear at this stage if the *OCT4* positive cell population has any pluripotent characteristics.

We chose two archetypal stem cell markers as the focus of this study, *OCT4* and *SOX2*. *OCT4* (*Oct3/4 or POU5F1*) is a member of the Oct-family of POU transcription factors. *SOX2* belongs to the *Sox* gene family, which are HMG box transcription factors that interact functionally with POU domain proteins. Both *SOX2* and *OCT4* play a key role in regulating stem cell pluripotency and differentiation. However, *SOX2* expression is associated with multipotent and unipotent stem cells, while *OCT3/4* is exclusively expressed in pluripotent stem cells. *SOX2* is expressed in adult neuronal stem cells and is retained in some populations of differentiating neurons [24–26], while the expression of *OCT4* in NSCs is unclear due to methodological problems [27]. However, a few studies show that Oct4 is expressed at both the mRNA and the protein level in “naïve” mouse [28–31] and human neurospheres [32], and that the expression drops during differentiation. The results of Massa et al. [32] based on qRT-PCR demonstrated dramatic decrease in *OCT4* expression in cortical neurospheres after the first week of differentiation. In contrast striatum derived neurospheres expressed the same level of *OCT4* before and after one week of differentiation [32]. This corroborates our qRT-PCR results where no significant difference in *OCT4* expression in neurospheres in GM and DM was detected. Immunostainings suggest that there were overall fewer *OCT4* positive cells but also fewer cells in total in neurospheres cultured in DM as compared with corresponding neurospheres cultured in GM (Figure 6). This supports the qRT-PCR data showing that overall the *OCT4* expression was not decreased when switching from GM to DM.

Functionally, hypoxia promotes proliferation and multipotentiality of CNS precursors [33] and promotes proliferation of human mesencephalic precursors [34]. *OCT4* expression may therefore be upregulated inside the neurosphere due to the low O_2_ tension inside neuropheres [35]. It has also been shown that *OCT4* is a specific target gene of hypoxia-inducible factor-2α (HIF-2α) [36], which may explain how stem cells may sense hypoxic conditions in their niches, and how low pO_2_ can modify stem cell function directly [37]. This is one possible explanation to the expression of *OCT4* in the cells located in the center of neurospheres. However, 24h post transfection, *OCT4* (and *SOX2*) mRNA positive cells were located at the surface of the neurospheres indicating that sphere formation is triggering the induction of *OCT4* while low O_2_ tension might further increase or maintain the expression. The small number of cells positive for *OCT4* and *SOX2* mRNA respectively indicate that differentiation is suppressed inside the neurospheres. This was further more confirmed by immunostaining with TH specific antibodies, which showed no staining of the cells in the center of the neurospheres (Figure 7) but strong staining in the cells in the periphery of the neurospheres.

Neurospheres cultures represent a heterogeneous system with respect to the size of the clusters, and the proliferative, differentiation, and developmental potential of parental clone-forming cells. Suslov et al. [5] proposed a hypothetical model for the relationship between neurosphere size and the maturation level of clone-forming cells. Their findings suggest that the size of a clone might reflect responsiveness to growth factors and the proliferation/differentiation status of the parental clone-forming stem/progenitor cell. In this model, cells with varying developmental potential were proposed. Clone-forming cells able to give rise to neurospheres demonstrating different stages of maturation were distinguished and proposed to have a different arrangement within the neurosphere architecture. The most immature clonogenic cells was suggested to be localized in the core of the neurosphere, while progenitor and differentiated cells were located at the shell. This hypothetical model is in agreement with the experimental results shown here (Figures 2A, 2B, 4 and 7).

The relatively slow rate of proliferation and physiological senescence in culture make the use of human neural stem cells cumbersome under some experimental and pre-clinical settings. The immortalization of hNSC with the *v-myc* gene generated stem cells with enhanced proliferative capacity, which greatly facilitates the study of NSCs *in vitro* and *in vivo* [38]. *v-myc* increases the pool of self-renewing cells and shift towards a more primitive cellular identity but without affecting the differentiation marker expression. Thus *v-myc* does not block differentiation, but delays cell cycle exit [39]. Two different studies [40,41] aimed to show the relationship between *c-myc* and the core pluripotency factors *OCT4* and *SOX2*; *OCT4* and *SOX2* have common target genes but few of these genes were targets for *c-myc*. A functional distinction between the *myc* binding sites and the sites to which *OCT4* and *SOX2* binds to was also revealed [40]. None of the *myc* DNA binding sites could mediate transcriptional activity of OCT4 and SOX2 respectively, suggesting that *myc* functions in a way that is distinct from the other pluripotent factors [41,42]. Expression of *OCT4* in monolayer growing cells was not detected in the present study suggesting that the induction of the *OCT4* expression in neurospheres is likely not an artefact from *v-myc* overexpression in LUHMES but a consequence of the cell environment and 3D architecture of the neurosphere.

As described by Sholz et al. [43], several precursor cell markers including *SOX2* were examined in LUHMES at day 0 (undifferentiated) and at day 5/6 (differentiated). We could confirm that undifferentiated LUHMES expressed *SOX2* mRNA and that the cognate protein was expressed in the cytosol (Figure 6B) [43]. The results of MB *SOX2* (Figure 2A) and qRT-PCR (Figure 5) measurements supported immunocytochemistry data. The fact that the undifferentiated adherent LUHMES cells retain some phenotypic features usually associated with immature cells was also conﬁrmed by staining for polysialylated neural cell adhesion molecule (PSA-NCAM), which was positive on day 0 for all cells and still positive for some but not all cells on day 5 [43].

Small structures resembling the whip-like appendages called primary cilia were frequently observed in adherent non-differentiated LUHMES (Figure 3A and B upper bright field). Similar structures can been found on stem cells. LUHMES cells are *v-myc* immortalized and thus genetically altered and perhaps it is not the best representative cellular model for study normal gene expression involved in lineage analysis and fate determination of NSCs. However, the genuine multipotentiality of the source cells (human embryonic mesencephalon tissue), their strict growth factor dependence, fast neurosphere formation, and their rapid proliferation arrest after differentiation induction, make them a suitable model system for this study. Cells in neurosphere are regarded as multipotent in their ability to generate all major neuronal phenotypes [32]. However, it is not possible from the data presented here to conclude that the OCT4 mRNA positive cells inside LUHMES neurospheres have any multipotent or pluripotent potential. Such analysis requires that the LUHMES neurospheres are dissected and the Oct4 cells are purified and subsequently analysed functionally for multi- or pluripotency. Such analysis is challenging because LUHMES cells grown as neurospheres are very difficult to maintain and passage (data not shown). The expression of the OCT4 gene suggests that these cells have stepped back in maturity functionally. However, it is possible that the activation of Oct4 expression by 3D clustering of cells is unique to the LUHMES cells and not representative of a normal nerve stem cells.

The signal from the *SOX2*-MB showed fluctuations in the intensity during differentiation, and was halved at day six (Figure 3C). This indicate that the MB was able to recover from the “open” fluorescent to “closed” non-fluorescent state. However, the signal from the *SOX2*-MB did not disappear completely, suggesting that *SOX2* was still expressed in differentiated neurons. Data presented here and elsewhere [24,25,43], showed residual amount of SOX2 expression suggesting that molecular beacons can sense also down regulation of genes. We were able to detect an increasing number of *SOX2* and *OCT4*-positive cells in neurospheres grown in GM. This indicates that not all of the MBs in the intracellular pool s hybridized to *OCT4* and *SOX2* mRNA. The MB pool will be diluted after each cell division; however, as long as the pool of MB is larger than the pool of target mRNA, the MB will be able to emit maximum signal proportional to the amount of transcript in the cell. When the pool of MB inside the cell is smaller than the pool of target mRNA, the MB can emit a signal, but it will be weaker and not proportional to the true level of transcript. At some point, the MB will be too diluted and signal lost despite the cells expressing a high number of target molecules. It is possible that some of the *SOX2* and *OCT4*-negative cells in neurospheres grown in growth medium (Figures 2 and 3, respectively) are indeed *SOX2* and *OCT4* positive. Despite these limitations, transfected MBs remain a very powerful tool for use with cells that do not divide extensively, such as stem cells after induction of differentiation, while the use of MBs might be limited to transient analysis in rapidly dividing cells.

The use of MBs as a tool for following differentiation is uncommon, and there are further limitations. For instance, it is difficult to know how many beacons penetrate each cell during transfection, and also whether each cell takes up an equal amount of probes [44]. Furthermore, the dynamic range of MBs appears inferior to qRT-PCR, since *SOX2*-MBs could only detect a 2-fold reduction in *SOX2* mRNA expression in monolayers of differentiated LUHMES cells (Figure 3C), while qRT-PCR demonstrated a 20-fold decrease in *SOX2* mRNA levels (Figure 5). Compared to destructive techniques like qRT-PCR and immunostaining, MBs can be used to track individual cells and even subcellular expression patterns of specific endogenous mRNA over time. GFP–reporter constructs are widely used to track which cells express certain genes, but GFP-reporters are constructed by recombinant technology which is time consuming and can result in changes in the regulation of the particular gene loci. It is therefore possible that the GFP reporter construct may not accurately reflect the wild type endogenous expression. MBs employ a rapid methodology, which does not require lengthy, and sometimes impossible genetic modifications, since MBs measure cytoplasmic mRNA expression directly. However, they may inhibit translation or cause changes to the metabolism of the mRNA, leading to functional differences in cells transfected with MBs. However, in our experiments targeting *SOX2* and *OCT4* using MBs, we did not observe any kinetic differences in differentiation or proliferation compared to non-transfected cells.

## Conclusions

Molecular beacons towards *SOX2* and *OCT4* mRNA were successfully used to track gene expression in living cells. The results showed that the aggregation of cells into 3D structures is sufficient to change gene expression in LUHMES, in this case to express the OCT4 gene. The tracking of cells expressing OCT4 and SOX2 also showed that there is a spatial reorganization of the cell mass in neurospheres so that differentiated cells are located in the periphery and cells with stem cell like expression are localized in the center of the neurospheres. The molecular beacon technology employed here is relatively simple yet provides highly relevant results as the technique measure endogenous mRNA. The technique is adaptable to a wider range of application providing that cells survive the transfection procedure and the molecular beacons employed are specific.
